# Service Delivery Reforms for Asian Ageing Societies: A Cross-Country Study Between Japan, South Korea, China, Thailand, Indonesia, and the Philippines

**DOI:** 10.5334/ijic.4739

**Published:** 2021-04-06

**Authors:** Shinichiro Noda, Paul Michael R. Hernandez, Kyoko Sudo, Kenzo Takahashi, Nam Eun Woo, He Chen, Kimiko Inaoka, Emiko Tateishi, Wahyu Sulistya Affarah, Hamsu Kadriyan, Jun Kobayashi

**Affiliations:** 1 Bureau of International Health Cooperation, National Center for Global Health and Medicine, Japan, 1-21-1 Toyama, Shinjuku-ku, Tokyo 162-8655, JP; 2 Department of Environmental and Occupational Health, College of Public Health, University of the Philippines Manila, 625 Pedro Gil Street, Ermita, Manila, PH; 3 National College of Nursing, National Center for Global Health and Medicine, Japan, 1-2-1 Umezono, Kiyose-shi, Tokyo 204-8575, JP; 4 Graduate School of Public Health, Teikyo University, 2-11-1 Kaga, Itabashi-ku, Tokyo 173-8605, JP; 5 Yonsei Global Health Center, Yonsei University, Unit 415, Chang-Jo Gwan, Wonju-city, Gangwondo, 220-710, KR; 6 School of Public Administration and Policy, Renmin University of China, No. 59, Zhongguancun Street, Haidian District, Beijing, CN; 7 Graduate School of Nursing Science, International University of Health and Welfare, 4-3 Kozunomori, Narita-shi, Chiba-ken 286-8686, JP; 8 Division of Planning, Coordination and Information, Okayama Bizen Public Health Center, 1-1-17 Furugyocho, Naka-ku, Okayama-shi, Okayama 703 -8258, JP; 9 Faculty of Medicine, Mataram University, Jl. Majapahit No. 62, Gomong, Selaparang, Kota Mataram, Nusa Tenggara Barat. 83115, ID; 10 Department of Global Health, Graduate School of Health Sciences, University of the Ryukyus, 207 Uehara, Nishihara-cho, Nakagami-gun, Okinawa 903-0213, JP

**Keywords:** service delivery reforms, healthcare service delivery, integrated care, ageing societies

## Abstract

**Introduction:**

Japan’s health policies to address the most advanced-aged society have been the target of focus in Asia, but no studies have investigated this issue using tools for cross-country comparisons.

**Theory and methods:**

A cross-country study design was used to compare healthcare reform policies with a framework in Japan, Korea, Thailand, China, Indonesia, and the Philippines. Data were collected via document reviews and key informant interviews.

**Results:**

Three distinctions were identified. First, all countries except for the Philippines have policy decisions regarding reforms for the existing service delivery systems for healthcare, long-term care and welfare. Second, the most extensive service delivery reform is currently being implemented in Japan, whose system is shifting to primary health care. Third, the direction of the transformation of service delivery system is different between Thailand and China despite a similar level of ageing society. China has made progress on facility-based care integration between health and social care, whereas Thailand is focusing on home-based care.

**Conclusions and discussion:**

Doctor and hospital-based healthcare delivery system requires more drastic reform for an aged society. This fact implies that strengthening primary health care is not only useful for current health issues but also an investment for the aged society near future in low- and middle-income countries.

## Introduction

In 2016, around 12.4% of the population of the Asia–Pacific region was aged ≥60 years. By 2050, this value is projected to increase to more than 25%, which is equivalent to 1.3 billion people [1]. Furthermore, the elderly population in East and Northeast Asia and Southeast Asia are estimated to account for 36.8% and 21.1%, respectively, by 2050 [1]. Data also show that the older population in low- and middle-income countries is growing faster than those in high-income countries [2]. By 2050, almost 80% of the world’s elderly population will be found in the less developed countries [1]. Therefore, these countries are projected to have an increased ageing society in which the gross domestic product per capita could be significantly lower than that of developed countries at the same stage of demographic transition [3]. Developments in medical technology are a major driving force of national health expenditure growth, and ageing could influence growth relative to medical advances [4,5]. Therefore, earlier policy decisions or long-sighted policies focused on health service delivery systems and their financing are required for countries that are facing and will have an ageing population in the near future [6].

Chronic diseases and multi-morbidity become more prevalent in ageing societies, thus requiring the transformation of healthcare service delivery systems that were predominantly designed for acute illnesses. In the United Kingdom, the late 1980s saw a turn of events for the healthcare service delivery system aimed at community-based care, including the Griffiths report [7], the White Paper Caring for People [8], and the National Health Service and Community Care Act 1990 (the first piece of comprehensive legislation) [9]. Service integration represents another aspect of transformation of the healthcare service delivery. Various care models to address these issues have been established in developed countries. Among these, the chronic care model created by Wagner has been widely adapted as an integrated care approach in Europe and the United States [10,11,12,13]. The chronic care model predicts better functional and clinical outcomes via productive interaction between informed activated patients and prepared proactive practice teams demanding health system change in five components: self-management support, delivery system design, decision support, clinical information systems and community resources.

In 2002, the World Health Organization developed Innovative Care for Chronic Conditions by adapting the chronic care model for low- and middle-income countries [14]. Since 2008, primary health care has been revitalised, and it is recommended that an integrated, people-centred approach is employed in the development of healthcare service delivery systems to address ageing and chronic conditions [15,16]. This recommendation is based on experience and evidence obtained from different regions, including the European region, which previously indicated that integrated care is a principle for a new healthcare service delivery system for the elderly [8]. In a cross-case analysis involving Australia, Canada, the Netherlands, New Zealand, Sweden and the United States, Wodchis et al. reported that integrated care programmes involved bottom-up innovations driven by local needs [17].

In Asia, Japan implemented two service integration efforts: “care managers” as service coordinators and “a district support centre” for comprehensive services provided by chief care managers, public health nurses, and social workers aimed toward integration between long-term care services and preventive public health services in 2005. These two efforts were accompanied by or responded to the results of the long-term care insurance that was introduced in 2000. Despite the implementation of these efforts for the demand side, there were more serious issues in the super-aged, population-declining society, such as a continuing growth of healthcare and long-term care expenditure and a shortage of elderly care facilities and caregivers projected in the coming 10 years. These require further policy decisions to address the fundamental problems of the Japanese healthcare service delivery system, which is heavily dependent on hospitals and highly specialised and subdivided professionals and fails to respond to demands of the elderly with multi-morbidity and disabilities. Then, Japan has established its own healthcare service delivery model, known as the community-based integrated care system [18].

The Government of Japan introduced the new model to Asian countries, including low- and middle-income ones in response to the demand that they learned from the most advanced-aged population in the world [19]. However, since the base of the health system significantly differs between Japan and any other countries, it was not easy for Asian countries to comprehend the model. Primary health care is a community-based healthcare service delivery system first described in the Alma-Ata Declaration of 1978 and is the basis of the health systems for most low- and middle-income countries; however, Japanese health systems are not based on it. It would be useful for other low- and middle-income countries to understand the differences in healthcare service delivery reform policies for the ageing society between Japan and other countries. To date, no studies have investigated using a framework to allow cross-country comparisons. Hence, the present study compared healthcare service delivery reform policies for the ageing society in Japan, Korea, Thailand, China, Indonesia and the Philippines.

**Figure 1:**
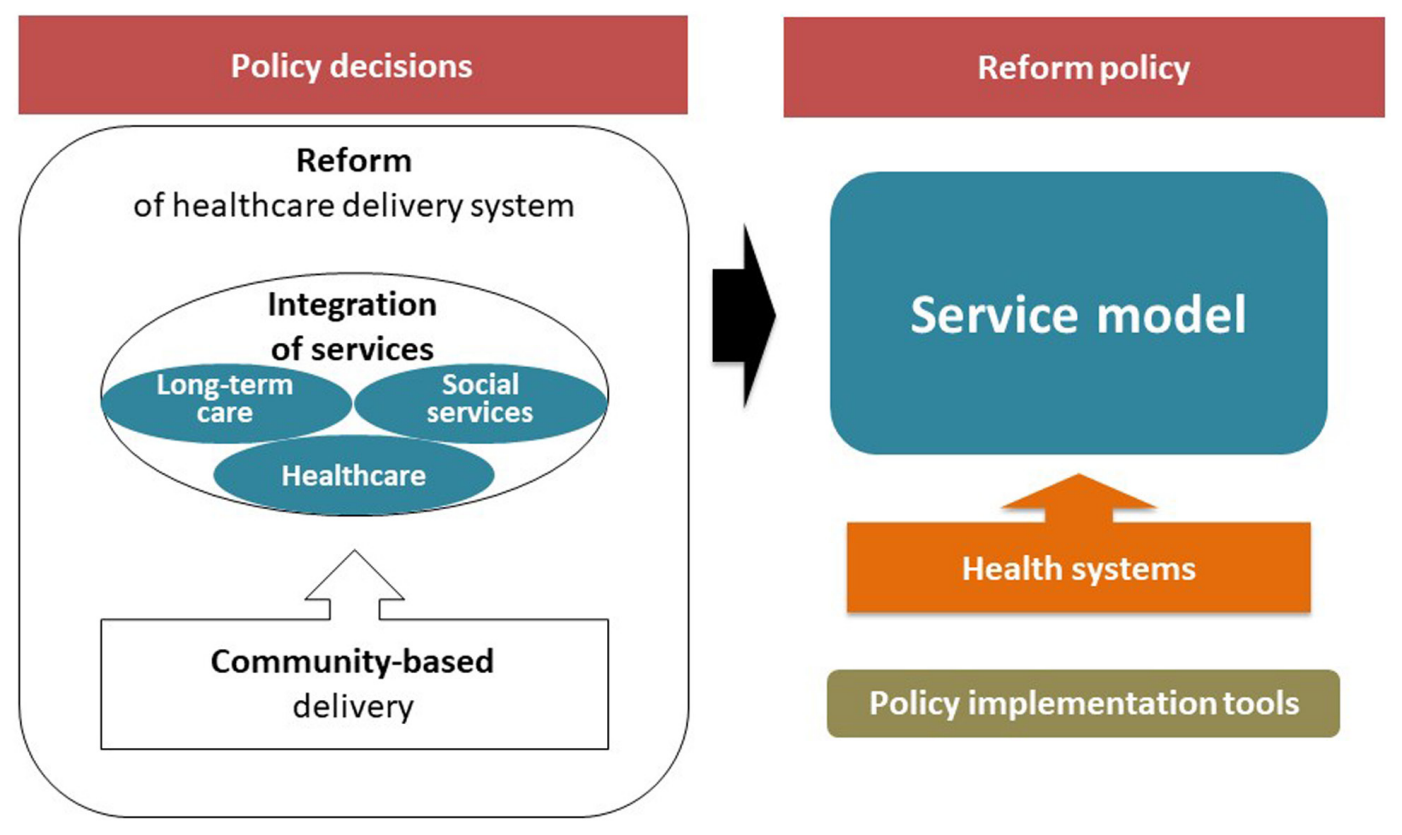
A conceptual framework developed to compare policies.

The present study used a cross-country study design. We developed a conceptual framework, illustrated in ***Figure 1***, to compare policies from the study countries. The framework comprised two aspects: policy decision and reform policy. For the policy decision on the reform of healthcare service delivery for the elderly, we considered three areas: whether there was any policy decision to undertake a reform of existing healthcare service delivery systems; whether the reform included integration of services between healthcare, long-term care and welfare services; whether it aimed to achieve a more community-based service delivery. In this study, “Integration of services” means that healthcare services are delivered in coordination with long-term care and/or welfare services and is equivalent to “integrated care.” In terms of the reform policy, the study compares the policies in three areas, namely, service model, health systems and policy implementation tools. For the service model, we compared the types of services included and the modes of delivery in the model that is suggested as a new healthcare delivery system for the elderly. For the health systems, financial sources for the implementation of a model and the introduction of new type of workforces, new information system, and coordination mechanism to operate the model were described [20]. The policy implementation tools considered included laws, strategy, programmes, budget, incentive, and monitoring and evaluation [21].

Data were collected via document reviews and key informant interviews using 25 question items based on the conceptual framework (***Table 1***). Document reviews were performed on relevant policies, strategies, regulations and decrees for each of country under study. Relevant reports and scientific articles were also included. Key informant interviews were conducted with core health officials of related ministries such as, the Ministry of Health, from which study directly obtained several documents. For the second study covering Japan, Korea and Thailand, key informant interviews were conducted with health officials of provincial health and welfare departments and district health and welfare offices as well as service providers such as doctors and long-term caregivers at the district level to determine the factors that promote and hinder the implementation of reform policies. Information collected for the second study was also used.

**Table 1:** Question items.


ASPECTS	AREAS	NO.	QUESTION ITEMS

Policy decisions	Reform	Item 1	Is there any policy decision to reform current healthcare service delivery system to adapt to the ageing society?

	Integrtion	Item 2	Does the healthcare service reform policy aim at integration of healthcare and long-term care?

			Does it aim at integration of healthcare and welfare services?

	Community-based	Item 3	Does it aim at more community-based care?

Reform policy	Service model	Item 4	Name of service delively model

		Item 5	Services (What kind of services are provided through the model?)

		Item 6	Unit of a service delivery system (Geopgraphical area in which a delivery system of the model is established. E.g. adminisrative unit, school unit, etc)

		Item 7	Models referred (Which service delivey models were used as a reference to develop the service delivery model?)

		Item 8	Who developed the model?

	Health systems	Item 9	Type of financial source for the services Self help: individual & family, Mutual help: informal network based on locality or co-belongingness, Social solidarity: insurance, Public assistance: tax [22]

		Item 10	New special heatlh workforces introduced for the model

		Item 11	New special other workforces introduced for the model

		Item 12	New information system introduced for the model

		Item 13	New coordination mechanism introduced to facilitate collaboration between health care service providers and welfare service providers

	Policy implementation tools	Item 14	Name of the policy document to introduce the model

		Item 15	Issuing body/institution

		Item 16	Date of officially approved

		Item 17	Policy goal

		Item 18	Policy objective

		Item 19	Policy timeframe

		Item 20	Name of the strategies

		Item 21	Name of the laws or decrees

		Item 22	Name of the program

		Item 23	Source of finance for the program

		Item 24	Incentive to implement the policy

		Item 25	M&E system to check implementation status of the policy


Ethical clearance for the study was obtained from the National Center for Global Health and Medicine Ethical Committee, Japan (approval number 2136).

## Description of Policy Development

### Policy decision on the reform of current service delivery systems for healthcare, long-term care and welfare

Japan, Korea, Thailand, China and Indonesia have issued policy decisions on the reform of existing service delivery systems for healthcare with consideration of integration with long-term care and welfare as well as more community-based delivery, whereas the opposite is true for the Philippines (***Table 2***).

**Table 2:** Policy decisions on reform of healthcare delivery.


QUESTION ITEMS	JAPAN	KOREA	THAILAND	CHINA	INDONESIA	PHILIPPINES

Is there any policy decision to reform current healthcare service delivery system for ageing?	Yes	Yes	Yes	Yes	Yes	No

Does it aim at integration of healthcare and long-term care?	Yes	Not yet	Yes	Yes	Yes	Not applicable

Does it aim at integration of healthcare and welfare services?	Yes	Yes	Yes	Yes	Yes	Not applicable

Does it aim at more community-based care?	Yes	Yes	Yes	No	Yes	Not applicable


Long-term care insurance was introduced in Japan in 2000. After a decade of implementation, three directions were recommended by the National Commission on Social Security Reform: from hospital to community-based care; from cure to cure and care; and comprehensive reform of medical care and long-term care. Thus, one of the policy objectives in the Comprehensive Reform of Social Security and Tax that was approved in the 2012 diet was the establishment of a community-based integrated care system by 2025.

In Korea, a long-term care programme was implemented in 2008 to support physical activities and household chores for elderly people who face difficulties in daily life and provide services to those aged ≥65 years (National Health Insurance System, 2014). The Ministry of Health and Welfare announced plans to introduce a Community Care Service Program on November 20, 2018. The outline of the plan was that senior citizens stayed at home or in group homes instead of hospitals or nursing homes and received care from the local community. The ministry plans to initiate 12 pilot programmes over 3 years from 2019 and implement a nationwide programme.

In Thailand, the National Commission on the Elderly and the Ministry of Social Development and Human Security revised the Second National Plan on the Elderly (2002–2021) in 2009, stating the need to ‘establish and develop health and social service including the long-term community-based care fully accessible to and usable by the elderly by emphasising the home care model inclusive of healthcare and social service simultaneously’. The model addressed the following services: (1) a service that supports long-term care; (2) a nursing system; (3) treatment of significant chronic diseases, such as hypertension, diabetes, and cerebrovascular disease; (4) community-based volunteers; (5) support to caregivers giving access to knowledge and skills appropriate to elderly care.

In China, the following directions on transformation of the healthcare service delivery system in the ageing society are included in policy statements: to strengthen the ‘gatekeeper’ role of community health facilities; to establish geriatric departments at secondary-level hospitals; to provide basic public health package, including management of health, hypertension and type 2 diabetes, and Chinese traditional medicine for older persons; to encourage provision of continued health services, including in-patient, rehabilitation, daily life care, and hospice care; and to encourage integration of health and social care. Furthermore, in 2013, the State Council of China issued statements on accelerating development of the elderly care industry as well as statements on promoting the development of the health industry, including establishment of an integrated health and social care system conforming to Chinese conditions, covering urban and rural areas. The integrated care system aims to achieve an appropriate scale and reasonable functions, provide comprehensive and continuous service, and improve primary health facilities to provide in-home services for the elderly. Furthermore, all the medical facilities are expected to establish a ‘green channel’ for older persons to provide convenient appointments and healthcare services and for all elderly care institutions to provide different forms of health services for older persons. The target year for these policy objectives is 2020.

Indonesia has developed policies to establish a National Commission for Elderly People with strategies to improve elderly welfare. These and successive policies have led to the development of community service centres for the elderly. The centres (known locally as posyandu lansia) integrate healthcare and welfare services for the elderly within the established healthcare system. The Ministry of Health and Welfare also developed the National Action Plan for the Elderly that aims to provide a reference for the central government, regional, and other stakeholders in the form of steps that must be continuously carried out to improve the health status among the elderly population by 2019.

However, despite the services that are provided for elderly people, there is still no policy decision on the reform of current service delivery systems in the Philippines.

### Reform policy

#### Service models

The service models in Japan, Thailand, China and Indonesia include healthcare, long-term care and social services for the elderly. The healthcare in these countries address most of the services that can be delivered by a general practitioner and medical staff, including emergency care, palliative care, and terminal care (cf. ***Table 3***). Integrated services are provided both at home and in facilities, such as nursing homes in the Japanese model, at home in the Thai and Indonesian models, and in long-term care facilities or community health centres in the Chinese model. In the Japanese model, home care mainly relies on private clinics, home visit nursing stations, and home-based long-term care agencies according to a care plan developed by a care manager working at one of these care facilities. On the other hand, district hospital teams provide integrated home care service in the Thai model and community service centre mobile teams in Indonesia. The Japanese and Thai models recommend health promotion activities via community groups or civil society organisations. Provision of rehabilitation at home is exceptionally limited in both Thailand and Indonesia.

**Table 3:** Services covered by delivery models in Asian countries.


COUNTRY	DELIVERY MODEL	SERVICES COVERED BY THE MODEL

Japan	Community- based integrated care systems	Home healthcare system Medical care provided in collaboration with varioushealthcare cadres; palliative care family support homecare arrangement support emergency home visit hospitalisation home terminal care terminal care at long-term carefacilities Long-term in-home care Home visiting long-term care home visiting nursing care day-care centre multifunctional long-term care services in a small group home short-term in-patient care welfare equipment 24-h in-home care service combined service of home visiting nursing care and short-term in-patient care Facility-based long-term care Nursinghome health facility sanatorium-type medical care facility group home Preventive long-term care Livelihood support

Korea	Reach in g-Out Community Service Centre	Welfare service: integrated welfare services Identifying welfare blind spots reaching-out servicesof welfare counselling (integrated case management, connection to necessary services, application to receive subsidies/benefits) integrated case management (childbirth/parenting, poverty caring, crisis family, elderly >65 years) searchingand investigating resource (local welfare community composition) Visiting nursing service Visiting nursing (visiting health check-up, health counselling) primary prevention activities (health education, exercise education, disease prevention activities, health programmes) association with medical institutions through ‘301 Network’, introducing the client to the public hospital

Thailand	Home care model	Continuous medical care service fromhospitalto community Services that support long-term care nursing system treatments for significant chronic diseases (hypertension, diabetes mellitus, cardiovascular disease) community-based volunteers support to caregivers Social services forthe elderly in the community Collaboration with the elderly club and the elderly school for prevention activities

China	Integrated health and social care	Atlong-term care facilities or the service area for acommunity health centre Social care Daily life care spiritual consolation, and cultural activities Healthcare Medicalconsultation preventr/e health seivice health e<amination disease diagnosis and management rehabilitative service; and hospice care

Indonesia	Community service centre for elderly (Posyandu lansia, Pos pelayanan terpadu, Puskesmas santun lansia)	Community service centre provides comprehensivecoverage of health mobile team services for elderly resident communityin a village Posyandu lansia Posbindu *Protection and activities: main rehabilitation* Regular nursing home: fulfilment of basic needs of elderly people living in a care home Day care: actualisation of elderly people living alone or with family via activities at the institution Home care: fulfilment of basic needs and mentoring neglected elderly KUBE/UEP (Kelompok Usaha Bersoma/Usaha Ekonomi Produktif): A government program which empowers poor groups providing business capitaVProductive economic enterprises increase in income and elderly income that can be earned by productivity ASLUT (Asistensi Social untuk Lanjut Usia Telantar): social assistance for neglected elderly

The Philippines	There is no health service reform specific for elderly care; however, the following services are provided by the government	Home healthcare system Medical care provided in collaboration with varioushealthcare cadres palliative care family support homecare arrangement support emergency home visit hospitalization home terminal care; and terminal care at long-termcare facilities In-home long-term care provided by thelocal government unit (i) Home visiting long-term care andhome visiting nursing care Facility-based long-term care viaresidential care facilities managed by the Ministry of Social Welfareand Development Livelihood support as provided by the Ministry ofSocial Welfare and Development via the Senior Citizen Centre


The current models in Korea and Indonesia provide integrated services for not only the elderly but also pregnant women and the poor via home visits by welfare officers and nurses based at public community service centres (cf. ***Table 3***). However, medical care services, such as diagnoses and treatments, are out of the scope of the Reach-Out Community Service Centre model of Korea because they are provided through existing integrated care system.

The Philippines lacks a healthcare reform policy for the elderly, however, healthcare and long-term care at home have been provided by local government health units. The Ministry of Social Welfare and Development is responsible for facility-based long-term care and livelihood support for the elderly people.

#### Health systems

All models mobilise various sources, such as tax, public health insurance, self-help, and mutual help to some extent (***Table 4***). The Japanese and Thai models introduced new workforces to operate the models. In the Japanese model, a livelihood support coordinator is a community health worker who is employed part-time and paid by a municipality. The coordinator has three main roles: local resource development, networking among stakeholders and matching the needs of elderly people and the available services of the model.

**Table 4:** Building blocks of the health systems that are introduced for implementation of the models.


COUNTRY	TYPE OF FINANCIAL SOURCES	NEW SPECIAL WORKFORCES	NEW INFORMATION SYSTEMS	NEW COORDINATION MECHANISMS

Japan	Self-help (individual and family) mutual help(informal network based on locality orco-belongingness) social solidarity(health and long-term care insurances) public assistance	Livelihood support coordinator	There is not a single standardised network system of patient and in-home care information, which is expected to facilitate information sharing among different care providers. However, many municipalities introduced a unique cloud-based network by themselves	Technical working group for individual cases, working group to identify common problems at community level, working group for policy formulation at municipal level

Korea	Public assistance (Ministryof Health and Welfare and Seoul Metropolitan Government Budget) Joint fund Private resources (religious, educational, civic qroups. etc.)	None	Using the existing welfare computer network, Scheduled to integrate information system between the Ministry of Health and Welfare and the Seoul Metropolitan City	Work with welfare serviceproviders (social welfare officers) and visiting nurses (working at publichealth centres) In the case of public health centres

Thailand	Mainly public assistance (tax for medical and welfare services, including prevention activities), social solidarity (Health insurances) Tambon Health Fund by the National Health Security Office Self-help (Out of pocket)	Elderly caregiver	None (theThai government plans touse the individual identification number to facilitate sharing of information, including medical records and income, welfare service usage, but thisis not only introduced forth is model)	The National Commissionon the Elderly underthe cabinet and new Ministry of SocialDevelopment and Human Security

China	Mainly self-help (individual and family) social solidarity (insurance), public assistance (tax) Seldom mutual help (informal network based on locality or co-belongingness)	None	The central policies encourage local governments to establish asingle information systemto facilitate information sharing among different care providers, but it is not mandatory. Some companies establish such informationsystems by themselves	collaboration contract between health and social care facilities for institutionalised persons and health and social care union; social care institutions to setup hospitals and nursing homes, which make the coordination an internal issues; community health centre to provide in-home medical, nursing and rehabilitation service

Indonesia	Self-help (out of pocket), social solidarity (Indonesia National Health Insurance), Public assistance (local government resources and Ministry of Social Welfare	None	Using the existing welfare computer network with improvement for electronic-based reporting (in Puskesmas, e.g., e- posyandu) There is no new information system for client-oriented welfare services	None

The Philippines	Self-help mutual help social solidarity public assistance through the Philippine Health Insurance Corporation	None	None	None


There is a requirement for a single information system that facilitates communication among care providers in the Japanese and Chinese models, and some local implementers of the models have voluntarily introduced a new information system. Each municipality establishes a community care meeting system, which is a coordination mechanism between health and welfare, as a subsystem of the Japanese model. The mechanism consists of a three-layer platform with different purposes: micro-level meetings to solve problems of individual cases, meso-level meetings to tackle community problems, and macro-level meetings to address municipality challenges. China also introduced multiple coordination mechanisms between health and welfare, both among and within facilities. While the Thai model established a national commission on the elderly, it did not introduce a special coordination mechanism among service providers. The Korean model encourages cooperation between home visiting nurses and welfare officers via educative sessions.

### Tools for implementation of the policies

The Japanese reform policy was translated into a national programme, known as the Community Support Program, with implementation guidelines for the departments of subnational health and welfare. The relevant laws were amended to give authorisation to the implementers. While this does not cover all activities of the programme, a special fund was established to support the programme at a subnational level. Financial incentives for providers of the services defined in the programme were introduced using a provider payment mechanism of the social health insurance and long-term care insurance. A subprogram of the Community Support Program was monitored using a set of indicators by each municipality with primary responsibility for the implementation. However, a national system to monitor progress of the community-based integrated care system has not yet been fully established.

The Korean ministry devised a pilot programme from 2019 and will also develop a nationwide community care programme. The main program includes integrated health and welfare services, including long-term care, housing or group home, public transportation services and enhancement of the local community capacity.

In Thailand, the home care model was implemented without any legislative actions or specific budget and incentives. It mainly relies on doctors, public health nurses and community health volunteers as well as family members of the primary health care system, with the capacity to build on this, such as by including training on non-communicable disease management.

China also implemented the policy without legislative tools. However, the Chinese government allocated part of the Central Finance and Welfare Lottery Public Welfare Fund to finance the development of integrated care. Some local governments provide financial support to develop integrated health and social care. Health insurance can cover part of the expenditure on health services. Long-term care providers generally have incentives to promote integrated care when they are able to have more customers and charge higher prices. Some hospitals and community health centres may have more revenue. However, the incentive for health workers in health facilities is not high in the majority of areas. The 90 national pilots of integrated health and social care are required to report their progress to the National Health Commission every 6 months. In addition, ‘The 13th Five-Year National Ageing Enterprise Development and Elderly Care System Construction Plan’ set indicators to monitor progress in integrated health and social care services.

A health minister’s regulation was issued for the National Action Plan for Elderly in Indonesia from 2016 to 2019. This aimed to develop community service centres for the elderly in villages, and funding was provided by the national and respective local government budgets. Additional funding may come from joint funds from non-governmental organisations and financing from private establishments as a part of their Corporate Social Responsibility. There are financial incentives for the programme managers and cadre (village health workers). Furthermore, top performing community health service centres are recognised (puskesmas santun lansia award). Monitoring and evaluation of the strategy indicators are performed by all levels from central (Ministry of Health) to village (local government units).

## Discussion

We aimed to reveal differences in healthcare service delivery reform policies for the ageing society between Japan and those of other countries. We compared six Asian countries with different extents of ageing.

Our comparison revealed three distinctions. First, Japan, Korea, Thailand, China and Indonesia have policy decisions on the reform of the existing service delivery systems for healthcare, long-term care and welfare, whereas the Philippines does not. Second, we found that the most extensive service delivery reform is currently being implemented in Japan. Third, the direction of transformation of service delivery system was different between Thailand and China, despite a similar level of ageing society.

The proportions of the elderly aged ≥65 years as of 2015 in Japan, Korea, Thailand, China, Indonesia and the Philippines are 26%, 13.0%, 10.6%, 9.7%, 5.1%, and 4.6%, respectively [23]. Japan, Korea, Thailand and China have long been considered an aged or ageing society, where the proportion of the elderly exceeded 7% of the total population. Indonesia will become an ageing society within a decade. Although the healthcare delivery systems in these countries differ, all of them have begun to reform such systems with the integration of healthcare services with long-term care and welfare services as well as community-based delivery because their systems are insufficient to respond to the demands of the elderly. It is predicted that the Philippines will not become an ageing society until 2030 [23]. This may be the primary reason why they have not yet made any policy decisions on transformation of health service delivery system. When Japan became an ageing society in 1970, they also had not had specific health policy decisions for the elderly population until the first policy was the free elderly healthcare service in 1972, which caused a moral hazard among both healthcare providers and patients and was abolished in 2002. The Philippines currently cares for its elderly using their existing healthcare system, which is based on primary health care.

Among the five countries with a reform policy on healthcare delivery, the most extensive one is currently implemented in Japan. The proposed service model covers medical care, nursing care, long-term care, rehabilitation, and livelihood support both at facility and at home. The Korean reform policy includes preventive healthcare and welfare services but medical care and long-term care. Conversely, the Chinese reform policy covers a wide range of services from medical care to social care. However, it focuses on long-term care facilities and community health centres. The reform policies of Thailand and Indonesia also encompass a variety of necessary services for the elderly. However, in contrast to Japan, the two countries have not introduced new resources or subsystems such as new special workforces, new coordination mechanism among various service providers and stakeholders, legislative actions or specific monitoring systems for the implementation of the reform policy in terms of health systems and policy implementation tools.

The comparison revealed the different directions in terms of reform between Thailand and China despite the similar levels of ageing society. China has made progress on facility-based care integration between health and social care, whereas Thailand has focused on home-based care. Several factors can influence this difference. District hospitals in Thailand are being built within a well-established primary health care system as a core facility of the district health system. Nurses with additional postgraduate training in hospitals take a pivotal role for community health in cooperation with health centers. Various types of paramedical personnel with a bachelor degree have been produced to address emerging health issues within the district health system [24]. Integration and community-based delivery of services for the elderly people must not have been unpredictable demands. In China, the Ministry of Civil Affairs is responsible for the management of elderly care facilities. It has driven efforts to push for the integration of health and long-term care, together with the National Health Commission. However, after the new round of function modification between government sectors in recent years, the National Health Commission takes more responsibilities for integrated social and healthcare. These uncertainties between various sectors impede the development of integrated care to some extent. Community and home-based elderly care has long been underdeveloped in China. The community health facilities are under immense pressure to provide healthcare and basic public health services to reduce their capacity to provide integrated health services, such as in-home and preventive healthcare [25]. Additionally, in China, many people bypass secondary-level hospitals in favour of tertiary-level hospitals, leading to a very low bed occupancy rate in secondary-level hospitals [26]. Some of these hospitals are changing their function to long-term care to address the increasing demands of elderly people, which also promote the development of facility-based integrated care [27].

Korea is about to become an aged society with an elderly population >14%. It has already introduced long-term care insurance [28]. The current elderly care service transformation comprises integration between the preventive and welfare services and does not involve the medical care services. This is similar to ‘the district support centre’ phase that existed in Japan in 2005. Korea also needs to prepare for an increasing demand for long-term care services as well as control health and social care expenditure. In 2018, they began discussions to reform their healthcare delivery system [29]. The Korean healthcare service delivery system is relatively more community-based, with devolved community healthcare services to public health nurses, such as primary health care compared with the Japanese system. Therefore, Korea may not require such a drastic reform as Japan.

Primary health care first described in the Alma-Ata Declaration of 1978 works on the premise that there are limited resources, particularly the health workforce, to address health problems. Primary health care was later identified as a foundation to strengthen health systems, particularly in low- and middle-income countries. Although assessing the impact of primary health care on population health is challenging, systematic reviews have shown that primary health care has a positive role on population health in the long term [30,31]. The same study also presented the proposed framework by the Primary Health Care Performance Initiative, in which the health systems in low- and middle-income countries should become equitable, efficient, responsive and resilient. This will lead to improvements in health status [32]. The primary care setup among low- and middle-income countries practising primary health care may be maximised and eventually reoriented towards integrated care. A systematic review by Dudley et al (2011) showed that integration in low- and middle-income countries may improve both the rate of utilisation and the outputs of the healthcare system [33]. Although only interventions that involved integration of vertical programmes into general health services at the point of delivery were included in the review, it identified possible integration strategies: (1) addition of a health service to a communicable disease programme, such as educating mothers on family planning during their visit to immunise their child; (2) combining two or more services, such as management of sexually transmitted infections and family planning; and (3) development of packages targeting a specific population, such as Integrated Management of Childhood Illness. The third type of integration can be applied to the elderly population. Note that all these integration strategies have the healthcare facility as the point of service delivery. Some of the elderly may be non-ambulatory; therefore, it is necessary to include the elderly individual’s home, and even the community, as a point of care.

In the present study, the transformation of healthcare delivery system for the elderly was promoted by strengthening the capacity of the existing health workforce in Thailand, Indonesia and the Philippines, where the service delivery system is based on primary health care. Conversely, Japan has embarked on a drastic reform of the doctor and hospital-centred delivery systems. In fact, the current reform is the first ever reform of the Japanese healthcare service delivery system. Similar efforts have been made in Western Europe, in which the chronic care model, which aims for changes in the health system and team approaches as well as community resources, was developed and spread [34], despite the controversial results of cost-effectiveness evaluations [35,36,37,38,39,40]. What does the difference between middle and high-income countries imply? Does it mean that high-income countries can design a more comprehensive policy and implement it in a sophisticated manner? The answer is no. The difference should be regarded as a result of using existing healthcare systems to formulate and implement the same policy that aims for integrated and people-centred care services for the elderly. Although the high-income countries required a more drastic reform of the existing healthcare system, which is the doctor and hospital-centred systems, middle-income counties could take advantage of primary health care, which is a community-based healthcare service delivery system. The rural healthcare system in China was formerly one of the models of the primary health care initiative promoted with the Alma-Ata Declaration of 1978 [41]. However, during the next decades, China reformed the urban and rural healthcare system by introducing market-based system resulting in a more hospital-based delivery system. Now China appears to confront the dilemma that, despite the efforts made by Chinese government, the community and home-based integrated care for the elderly are still largely under developed. Primary health care and chronic care models for the elderly have failed to provide solid evidence of cost-effectiveness or cost-saving. Reforming a health service delivery system in an aging society is challenging itself. Thus, a policy option, such as reform based on community-based and integrated care that highly prioritizes to “access” and “quality” rather than “cost,” would be reasonable. In addition, a major driving force of national health expenditure growth in high-income countries is the developments in medical technology [4,5], which are primarily used in secondary and tertiary hospitals. Therefore, investing in primary health care that relies more on appropriate technology, in general, defined as small-scale, decentralized, people-centered, labor-intensive, energy-efficient, environmentally sound, and locally controlled [42], has potential for cost-saving.

The present study compared the healthcare service delivery reform policies for the ageing societies in Japan, Korea, Thailand, China, Indonesia and the Philippines by reviewing policies and other relevant documents and key informant interviews. Since the study was primarily concerned with content analysis of the policy, only current reforms were considered that were documented without depicting the entire existing service delivery systems for the elderly. As with any intervention, it is necessary to monitor and eventually evaluate how these policies are implemented at national, regional, community and end-user levels. We will evaluate the promoting factors for implementation of the reform policies in Japan and Thailand and report these findings in the future. Notwithstanding the limitations, the study is helpful for other countries that are going to be or in the early stage of an ageing society for the formulation of long-term policies focused on health service delivery systems.

## Conclusion

This cross-county study identified three distinctions between the policies for healthcare delivery transformation for elderly care in Japan, Korea, Thailand, China, Indonesia and the Philippines using the conceptual framework that not only considers the proposed service delivery model but also the other health system building blocks as well as policy implementation arrangement. First, all countries except for the Philippines have policy decisions on the reform of the existing healthcare delivery system. These initiatives aim to integrate such systems with long-term care and welfare services as well as to shift to more community-based delivery system. Second, Japan’s reform policy is the most extensive. Third, the direction of the transformation of the healthcare delivery system differs between Thailand and China, despite the similar level of the ageing society. The last two distinctions reflect the existing health system conditions, especially whether the system is based on primary health care or not.

The service delivery system of the most advanced-aged country in the world is shifting from the doctor and hospital-centred system to the integrated, community-based system, which requires more extensive reform than the other study countries whose healthcare delivery system is based on primary health care. This fact implies that strengthening primary health care is not only beneficial for current health issues but is also an investment for the future in low- and middle-income countries.
